# Pharmaco‐metabolomics opportunities in drug development and clinical research

**DOI:** 10.1002/ansa.202000178

**Published:** 2021-09-30

**Authors:** Prasad Phapale

**Affiliations:** ^1^ European Molecular Biology Lab Metabolomics Core Facility Heidelberg Germany

**Keywords:** clinical research, drug development, drug response, high‐resolution mass spectrometry, Indian Pharma, LC–MS/MS, metabolomics, personalised medicine, pharmacokinetics, Pharmaco‐metabolomics, triple quadrupole mass spectrometry

## Abstract

Pharmaco‐metabolomics uses metabolic phenotypes for the prediction of inter‐individual variations in drug response and helps in understanding the mechanisms of drug action. The field has made significant progress over the last 14 years with numerous studies providing clinical evidence for personalised medicine. However, discovered pharmaco‐metabolomic biomarkers are not yet translated into clinics due to a lack of large‐scale validation. Integration of targeted and untargeted metabolomics workflows into pharmacokinetic analysis and drug development can advance the field from bench to bedside. Also, Indian pharmaceutical research and its bioanalytical infrastructure are in a position to take on these opportunities by addressing challenges such as appropriate training and regulatory compliance.

## INTRODUCTION

The developments in mass spectrometry (MS)‐based metabolomics technologies can replace the present medical practices of limited chemical analyses with far more comprehensive metabolite profiles. These metabolic profiles (metabotypes) of an individual when combined with medical and multi‐omics (genomics, proteomics) information can be used to diagnose and monitor disease conditions, as well as understand its pathogenesis. Also, ‘metabotypes’ obtained before and during drug exposure can provide insights into the mechanism of drug action and predict inter‐individual variation to drug treatment.^1^ Since only 25% to 60% of the patient population show the desired response to the majority of drugs, there are over 2 million adverse drug reactions reported each year with 100,000 deaths in the United States and is the reason for up to 5% of hospital deaths in India.^2^, ^3^ This has raised critical research initiatives on developing personalised drug therapy worldwide.^4^, ^5^ The emerging branch of metabolomics called ‘Pharmaco‐metabolomics’ (PM) deals with challenge of prediction of inter‐individual differences in drug response based on patient's pre‐dose ‘metabotype’.^6^, ^7^, ^8^

The PM has been introduced in 2006 as a proof of principle study to predict personalised drug response of paracetamol in rats using their pre‐dose urinary metabolite profiles.^9^ In 2010, our group reported one of the first human PM studies to predict inter‐individual variation in pharmacokinetic profiles (PK) of immunosuppressive drug tacrolimus which is used during organ transplantations. We integrated pre‐dose urine metabolomics and drug response using multivariate statistics and metabolic network analysis to obtain metabolic phenotypes (represented by four metabolite markers) predictive of tacrolimus drug response.^10^ Since then PM research has made considerable progress in predicting the interindividual drug response variations which may not be well explained by their genotypes (Pharmaco‐genomics).^6^, ^11^ Recently, metabolomic data has been used to guide genomic information to provide insights into personalised drug response in so called ‘PM‐informed pharmacogenomic’ approach.^12^ This has led to the PM Research Network of 15 research institutes which performed several studies in the last decade.^1^, ^6^, ^13^ PM has shown its potential in xenobiotic toxicity assessment,^14^ patient segregation (responders vs non‐responders), understanding the mechanism of action of novel drugs.^1^ Moreover, some of the PM biomarkers are now becoming part of FDA (Food and Drug Administration) regulatory submissions.^6^, ^15^ Thus PM can be a promising prognosis tool for precision medicine to inform physicians about drug efficacy and possible adverse effects prior to drug therapy.

Despite its potential and progress the PM research has not yet provided validated clinical biomarkers for patient segregation (drug responders vs. non‐responders) or warn us of possible adverse drug reactions similar to pharmacogenomic warning labels for certain drugs.^6^, ^16^ In order to move the field forward large‐scale adoption of PM methods in routine bioanalysis is necessary. Hence it is proposed to perform metabolomic investigations not only during clinical trials but also in early drug discovery and development phases.^17^

Metabolomics in principle is small molecule bioanalysis similar to drug analysis, using liquid chromatography (LC)–MS as one of the major analytical platforms.^1^ PM workflows consist of pharmacokinetics and drug metabolism (DMPK) analysis which is then correlated with biofluid pre‐dose metabolomic profiles.^10^ Thus most of the PM analysis can be performed from the same biofluid samples using similar or modified LC–MS methods. One of the challenges in performing these two analyses from the same samples is optimizing the workflows to best utilise the instrument capabilities with smart data acquisition. PK bioanalysis is mainly performed on triple‐quadrupole mass spectrometers (QqQ) due to their sensitivity, specificity, and dynamic range. Drug metabolism studies at the early drug development stage required high‐resolution MS (HRMS) to characterise known and unknown drug metabolites. These both QqQ and HRMS methodologies are similar to the targeted and untargeted metabolomics workflows respectively. This perspective proposes to combine DMPK analysis with PM analysis in clinical and drug development labs. Further, perspective is given on India's large bioanalytical capabilities which can provide PM analysis as a research and service infrastructure to advance drug research.

## 1 SAMPLE COLLECTION AND PREPARATION STRATEGIES

**FIGURE 1:**
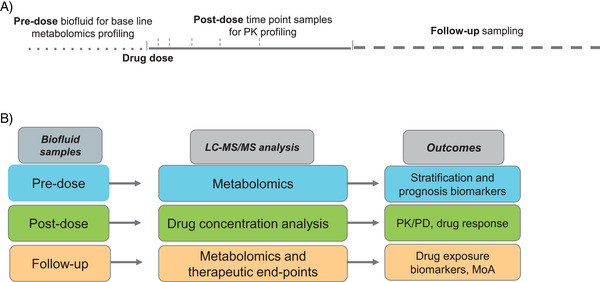
(A) sample collection scheme describing pre‐dose, post‐dose and follow‐up sample collection for respective analysis, (B) overview of Pharmaco‐metabolomics study and analyses performed during drug development and clinical research (MoA, mechanism of action; PK/PD, pharmacokinetics/pharmacodynamics)

In clinical and animal drug development studies, biofluids (e.g., blood, serum, plasma, urine, saliva and stool) are most commonly utilised due to their relative ease of collection, preparation, and storage. As shown in Figure 1A, the sampling scheme can be divided as 1) pre‐dose sample collection 24 to 48 h prior to drug dose, 2) post‐dose time points as per PK modelling (usually spread over 0.5 to 12 h) and 3) follow‐up samples (usually after 48 h post‐dose) to monitor the progression of drug response and therapeutic effect (pharmacodynamics) over a longer time. These samples can be divided for drug and metabolomic analysis.^13^ The sample storage at –80°C is often required before the bioanalysis. The study design should consider the stability of labile metabolites during sample treatment and storage. Also, sample treatments such as EDTA/Heparin anticoagulant during blood collection can cause interference in MS analysis. Such matrix effects should be assessed with appropriate blank control samples. The sample preparations can be common to DMPK and PM analyses depending on the type of biofluid samples and drug molecules under the study. Usually, the aqueous organic solvents (methanol and acetonitrile) are used for protein precipitation as well as metabolite extraction which saves a great deal of sample collection efforts and analysis time. However, the types of biological samples and analytes (drug or metabolite biomarkers) being studied are always a critical component in any experimental design. Based on the complexity of the biological matrix, extraction efficiency of drugs, and required metabolome coverage additional sample clean‐up such as liquid‐liquid extraction, solid‐phase extraction can be employed. In any case, the selected sample preparation, storage, and treatments method need to be validated for both drug and metabolomics analysis.^18^ The availability of a large number of clinical samples can be a major limitation for PM biomarker discovery and validation. Hence the proposed approach of utilizing samples from PK studies for simultaneous drug and metabolomics analysis can significantly improve the number of samples available for PM analysis. Although access to clinical samples is resource‐dependent, the workflow described here can partially overcome this challenge by developing LC–MS‐based analytical methods to profile drug and endogenous metabolites from the same samples.

## 2 PK AND TARGETED METABOLOMICS USING QqQ

The major MS instruments for bioanalysis are QqQ and HRMS. The advantage of LC coupled with QqQ tandem MS (LC–QqQ–MS/MS) is their sensitivity and dynamic range compared to HRMS. The QqQ instruments provide the required specificity for drug analysis by monitoring unique parent and daughter ion pairs in multiple‐reaction‐monitoring scan mode. Thus, QqQ instruments comply with the regulatory requirements of accuracy, precision, specificity, and sensitivity for bioanalytical method validation. The method validation can be performed as per guidelines to establish limits of quantification and detection, dynamic range, recovery, matrix effect, and stability.^19^, ^20^ The current bioanalytical methods are however restricted to the measurement of a single drug analyte and its internal standard from biofluid samples. It should be noted that the same biofluid with similar sample preparation does contain a wealth of information about endogenous metabolites (metabolome). This information is largely discarded during PK bioanalysis. As proposed here optimization of bioanalytical methodology can be performed for targeted metabolomics either simultaneously, sequentially, or in parallel to PK drug concentration analysis. The existing LC–QqQ–MS/MS set‐up can be utilised to perform additional targeted metabolomics studies. The same instrument can be tuned to measure more than 100 biologically important endogenous metabolites.^21^ The multiple‐reaction‐monitoring selection can be optimised in two ways: 1) retention time scheduling of drugs and metabolites to reduce the scan time/cycle time and 2) LC flow can be split for parallel analysis with another triple quad tuned for metabolomics analysis along with regular drug analysis.

This approach will have a major research implication for PM research by optimization of resources during clinical and animal studies. Also, commercial bioanalytical labs that routinely perform bioavailability and bioequivalence studies can provide additional services to their clients. However, such approaches can have an impact on the drug analysis quality of PK studies. More importantly, such arrangements may not comply with FDA regulatory requirements particularly for clinical studies in Good Laboratory Practices (GLP) settings where methods are required to be validated for quantification of a single drug analyte.^15^ The more suitable approach can be sequential analysis on the same instrument with different methods to obtain additional targeted metabolomics datasets. These datasets can then be processed using previously reported data analysis workflow for the correlation of endogenous profiles with drug response.^10^

Some of the PM biomarkers reported for drug response prediction (from pre‐dose samples) and drug effect assessment (from post‐dose samples) are summarised in Table 1. These provisional biomarkers are not yet clinically applicable due to limited studies performed on small sample size. Also, previous researchers have used different technologies such as nuclear magnetic resonance (NMR) and gas chromatography (GC)–MS for metabolomic analysis, which can be harmonised with common LC–QqQ–MS/MS methods for inter‐lab comparisons.

**TABLE 1:** List of drugs investigated for drug response predictions and drug effect assessment. These reported drug response markers can routinely be analysed using triple‐quadrupole (QqQ) coupled with liquid chromatography and tandem mass spectrometry (LC–QqQ–MS/MS) workflow

		Pharmaco‐metabolomics markers or pathways			
Drug	Therapeutic category	Drug response prediction (Pre‐dose)	Drug effect (Post‐dose)	Analytical methods used for Metabolomics	Ref
Tacrolimus	Immunosuppressant	Acetyl‐arginine, cortisol, methyl guanosine, phosphoethanolamine		LC–MS	^10^
Methylphenidate	Attention‐deficit and hyperactivity disorder	Ceramide, phosphatidylcholine		LC–MS	^22^
Metformin	Type 2 diabetes		Vitamin B12, methylmalonic acid, homocysteine	LC–MS	^23^
Simvastatin	Statin	Amino acids	Amino acids and urea cycle metabolites	GC–MS	^24^
Trastuzumab‐paclitaxel	Breast and stomach cancer	Spermidine, tryptophan		QqQ/Qtrap	^25^
Capecitabine	Cancer chemotherapy	Fatty acids, choline phospholipids		NMR	^26^
Methotrexate	Cancer chemotherapy	Organic anions		GC–MS	^27^
Atenolol	Beta‐blocker	Amino acids	Amino acids	GC–MS	^28^
Busulfan	Cancer chemotherapy	Deoxycholic acid, chenodeoxycholic acid, and linoleic acid		LC–MS	^29^

Abbreviations: GC, gas chromatography; NMR, nuclear magnetic resonance.

However, QqQ based analysis is limited in coverage and will have inherent limitations of low MS resolution in separating isobaric metabolites. The next section will highlight HRMS analysis which can overcome these limitations to provide simultaneous drug and metabolomic analysis.

## 3 DRUG DEVELOPMENT AND UNTARGETED METABOLOMICS USING HRMS

Drug discovery is a process that screens and selects New Chemical Entities as potential drug candidates which have shown activity against a particular therapeutic target of the disease. However, their safety, toxicity, PK, metabolism, and distribution are not yet established which is studied during the drug development process prior to clinical trials. Although drug metabolism or biotransformation is an essential process for the elimination of xenobiotics from an in vivo system it can generate active species or undesired toxic metabolites.^2^ Hence it is crucial to study absorption, distribution, metabolism, and elimination (ADME) properties of drug candidates in order to assess their safety. ADME studies involve screening of New Chemical Entities for metabolic stability across species (human/rat/dog/mouse/monkey) in various matrices including microsomes/S9 fractions, cytosol, hepatocytes, plasma, tissue homogenates, and buffer systems.^2^ The measurement of biotransformation products (drug metabolites) from these ADME assays are commonly performed using HRMS coupled with LC (LC–HRMS–MS/MS) in full scan MS and data‐dependent MS/MS mode. The ability of HRMS analysis to survey possible drug metabolites in an untargeted manner made it an indispensable tool for the drug development process. The recent advances in HRMS technology have significantly improved the scan speed, sensitivity, and coverage which is in some cases comparable to the QqQ. Here proposed HRMS workflow can replace QqQ to perform analysis of drug and endogenous metabolites simultaneously which makes them best suited for PM studies.

However, unlike targeted QqQ analysis, untargeted metabolomics data analysis comes with challenges from feature detection to metabolite identification.^18^ Further for PM applications the data analysis workflows should be customised for drug and exogenous peaks along with endogenous metabolite profiles. This can be even more challenging compared to traditional metabolomics studies considering false positive annotations possible from drug metabolites, exogenous compounds, exposomes, and background ions found in biofluid samples. Hence optimization of workflows for blank control samples and complete medical history, co‐medications, lifestyle, diet information should be considered during data analysis to exclude the false positive metabolite annotations. Recent studies have used advanced data analysis methods such as mapping metabolite concentrations into integrative reaction networks,^30^ and using machine learning for metabolite feature selection and prioritization of biomarkers for clinical evaluation.^31^

We have recently optimised a workflow for the analysis of duloxetine drug and bacterial metabolites from the same samples using LC–Orbitrap–MS/MS with our EMB–MCF spectral library,^32^ other public databases^33^ and a quality control system.^18^ We were able to quantify duloxetine up to 1 nM concentration and simultaneously measure over 100 metabolites from the same bacterial samples treated with the drug (Metabolights^34^ study identifier MTBLS1319: Bioaccumulation of therapeutic drugs by human gut bacteria: duloxetine accumulation at different concentrations).^40^ Such PM workflows can be used in the drug development process to perform a three‐way analysis of drugs, drug metabolism, and metabolomics. Figure 1B summarises LC‐MS analysis methods and possible information obtained from collected samples. 1) pre‐dose biofluid analysis can provide baseline metabolic profiles which can be used for prediction of drug response prior to drug administration, 2) post‐dose sample time points are mainly collected for PK analysis and some of the time points are essential for metabolomics analysis to assess the effect of drugs on endogenous metabolism with reference to baseline pre‐dose profiles and 3) follow‐up sample analysis evaluates long‐term effects of medication both as pharmacodynamics endpoints and overall metabolic health of the subject.^10^ Metabolomic profiling of these samples along with post‐dose time points can have implications in assessing drug toxicity markers as well as understanding mechanisms of drug actions.^13^ The more general applications of untargeted metabolomics in drug discovery and preclinical research are comprehensively summarised in literature reviews.^17^, ^35^

## 4 PERSPECTIVE ON INDIAN PHARMACEUTICAL RESEARCH

Indian pharmaceutical industry has over 3000 companies and a network of about 10500 manufacturing facilities which contributes to approximately 20% of the global generics market with an estimated US$20 billion export revenue each year.^36^ There are over 100 bioanalytical facilities approved by the Drugs Controller General (India) to perform GLP controlled bioavailability and bioequivalence studies.^37^ Also, India has one of the world's largest talent pools of pharmaceutical professionals with rapidly growing bioanalytical expertise due to the increasing number of ANDA (abbreviated new drug applications) filings from India. Most of the world's leading global pharmaceutical companies such as Eli Lilly, Sanofi, AstraZeneca, Novartis, Bayer and Merck, GlaxoSmithKline and Bristol‐Myers Squibb have their clinical research centres in India. India has also become a favourite destination of contract drug discovery research and clinical trials due to its lower operational costs, recent regulatory reforms, and several logistic advantages.^38^ Consequently, bioanalysis for preclinical and clinical studies for global regulatory submissions are now routinely performed from India. This has led to the growth of the Indian MS market by 10% annually compared to the global rate of 6.7%.^39^ As per www.marketstudyreport.com, this growth rate is even higher than the dominant North American MS market, which is projected to grow at 7.5% in 2021. The global bioanalytical testing market is expected to reach a value of $4.56 billion by 2027, which can potentially accommodate PM analysis. The largest North American and European bioanalytical markets outsource services to more affordable counties such as India and China. Such outsourcing can give more boost to Indian MS analysis labs in the coming years which are equipped with high‐end LC–QqQ–MS/MS and HRMS instruments.

Apart from the above‐mentioned factors, the MS market is also propelled by an increase in academic research focussed on proteomics and metabolomics. The LC–QqQ–MS/MS segment saw the fastest growth and consists of a 54% share of the Indian MS market, largely driven by the pharmaceuticals, clinical, and food safety segments. Similarly, the HRMS capabilities in India are increasing due to increasing interest in omics research in academic as well as industrial drug discovery research.^18^

Considering existing MS capabilities and availability of clinical samples, the scope of these bioanalysis labs can be expanded beyond bioanalytical toward providing service infrastructure for PM research.

## 5 FUTURE PERSPECTIVE

PM and pharmaco‐genomics studies are increasingly becoming part of FDA Regulatory submissions globally.^1^, ^6^ Such studies have the potential to improve our understanding of drug mechanisms of action for new drug candidates and predict personalised drug response. As PM progressed, there are huge opportunities for bioanalytical laboratories in the research and service sectors. India with its established set‐up in pharmaceutical and clinical research is in a unique position to harness this opportunity. Recently developed academic omics labs can share their expertise with industrial research particularly in the areas of metabolomics data analysis, new analytical method developments and integration of metabolomics data at the system biology level. The recently mushroomed MS core facilities across national institutes can share their infrastructure and expertise with drug development research. Such collaborations will also make the PM data and tools more open access and shared across the research community using various data repositories and portals.^34^ Sharing of the findings including raw data will ultimately benefit both industry and academic research through community contributions and open science.

However, there are two major challenges in moving forward with the implantation of the proposed PM framework. One is a need for additional training for bioanalytical researchers in metabolomics methods, data analysis practices and optimization of lab resources to perform simultaneous drug and metabolomic analysis.^18^ The second major challenge is the translation of metabolomics biomarkers into clinics has to go through a major test of regulatory compliance and acceptance among the clinical community. This often requires large scale clinical validation of biomarkers to prove their utility and comply with regulatory submissions. Although both of these challenges are relevant globally, from the Indian perspective the first challenge can be addressed more urgently. The appropriate training of bioanalytical professionals can expand the potential of Indian MS laboratories beyond routine pharmaceutical analysis. The proposed integrated PM workflows can help in addressing these challenges.

## CONFLICT OF INTEREST

The authors declare no conflict of interest.

## Data Availability

The associated raw data mentioned in this article are available on the MetaboLights repository with study identifiers MTBLS1301, MTBLS1319 and MTBLS1861.
